# Risk of Fracture with Thiazolidinediones: An Individual Patient Data Meta-Analysis

**DOI:** 10.3389/fendo.2013.00011

**Published:** 2013-02-26

**Authors:** Marloes T. Bazelier, Frank de Vries, Peter Vestergaard, Ron M. C. Herings, Arlene M. Gallagher, Hubert G. M. Leufkens, Tjeerd-Pieter van Staa

**Affiliations:** ^1^Utrecht Institute for Pharmaceutical Sciences, Utrecht UniversityUtrecht, Netherlands; ^2^MRC Lifecourse Epidemiology Unit, University of SouthamptonSouthampton, UK; ^3^Department of Clinical Pharmacy and Toxicology, Maastricht University Medical CentreMaastricht, Netherlands; ^4^The Osteoporosis Clinic, Aarhus University HospitalAarhus, Denmark; ^5^Pharmo InstituteUtrecht, Netherlands; ^6^Clinical Practice Research Datalink, Medicines and Healthcare products Regulatory AgencyLondon, UK

**Keywords:** thiazolidinediones, fracture, individual patient data, meta-analysis, epidemiology

## Abstract

**Background:** The use of thiazolidinediones (TZDs) has been associated with increased fracture risks. Our aim was to estimate the risk of fracture with TZDs in three different healthcare registries, using exactly the same study design, and to perform an individual patient data meta-analysis of these three studies.

**Methods:** Population-based cohort studies were performed utilizing the British General Practice Research Database (GPRD), the Dutch PHARMO Record Linkage System (RLS), and the Danish National Health Registers. In all three databases, the exposed cohort consisted of all patients (aged 18+) with at least one prescription of antidiabetic (AD) medication. Cox proportional hazards models were used to estimate hazard ratios (HRs) of fracture. The total period of follow-up for each patient was divided into periods of current exposure and past exposure, with patients moving between current and past use.

**Results:** In all three registries, the risk of fracture was increased for women who were exposed to TZDs: HR 1.48 (1.37–1.60) in GPRD, HR 1.35 (1.15–1.58) in PHARMO, and HR 1.22 (1.03–1.44) in Denmark. Combining the data in an individual patient data meta-analysis resulted, for women, in a 1.4-fold increased risk of any fracture for current TZD users versus other AD drug users [adj. HR 1.44 (1.35–1.53)]. For men, there was no increased fracture risk [adj. HR 1.05 (0.96–1.14)]. Risks were increased for fractures of the radius/ulna, humerus, tibia/fibula, ankle, and foot, but not for hip/femur or vertebral fractures. Current TZD users with more than 25 TZD prescriptions ever before had a 1.6-fold increased risk of fracture compared with other AD drug users [HR 1.59 (1.46–1.74)].

**Conclusion:** In this study, we consistently found a 1.2- to 1.5-fold increased risk of fractures for women using TZDs, but not for men, across three different healthcare registries. TZD users had an increased risk for fractures of the extremities, and risks further increased for prolonged users of TZDs.

## Introduction

Thiazolidinediones (TZDs) have been demonstrated to improve insulin resistance when applied to patients with Type 2 Diabetes Mellitus (T2DM). TZDs exert their insulin-sensitizing actions either directly, by promoting fatty acid uptake and storage in adipose tissue, or indirectly, by means of altered adipokine release (Yki-Jarvinen, 2004). Recently, an association of rosiglitazone with risk of cardiovascular outcomes (Schernthaner and Chilton, 2010) has led to withdrawal of the drug in Europe and restricted access in the United States, but pioglitazone is still used in the management of T2DM. Beneficial effects of pioglitazone use have been reported in patients with a recent acute myocardial infarction (Erdmann et al., 2007) as well as in patients with non-alcoholic steatohepatitis (Sanyal et al., 2010).

The increased adipogenesis caused by TZDs is to the detriment of the genesis of bone-forming osteoblasts (Benvenuti et al., 2007; Grey, 2008). Several studies have found that TZD use leads to a decreased bone mineral density (BMD) and an elevated risk of fracture (Betteridge, 2011). Women who were exposed to TZDs for 3–4 months had significantly reduced BMD at the lumbar spine and hip in two randomized controlled trials (Glintborg et al., 2008; Grey et al., 2011). A meta-analysis from 10 randomized controlled trials (Loke et al., 2009) showed that the use of rosiglitazone and pioglitazone was associated with a significantly increased risk of fractures [Odds Ratio 1.45 (95% CI 1.18–1.79)].

Observational studies have provided some contrasting results regarding sex difference and the type of fractures (Betteridge, 2011). This may be due to differences in study designs, study populations, the recording/under-recording of certain fracture types, the use of co-medication, the type of patients that are prescribed TZDs, and other potential causes of bias that exist in observational research.

Therefore, the first aim of this study was to estimate the risk of fracture with TZDs in three different healthcare registries, using exactly the same study design. This allowed us to compare in a consistent manner the patient characteristics of TZD users, the use of co-medication, the occurrence of fractures, and the risks of fracture for TZD users between these registries. The second aim was to perform an individual patient data meta-analysis of these three studies on the risk of fracture in TZD users, stratified by fracture type, sex, and duration of use.

## Materials and Methods

### Data sources

This study used data from the British General Practice Research Database (GPRD) which is part of the Clinical Practice Research Datalink, the Dutch PHARMO Record Linkage System (RLS), and the Danish National Health Registers.

The GPRD comprises computerized medical records for over 10 million patients under the care of general practitioners (GPs) in the United Kingdom (UK). The data recorded in the GPRD since 1987 include demographic information, prescription details, clinical events, preventive care provided, specialist referrals, hospital admissions, and their major outcomes. In the UK, the GP typically manages the prescribing for chronic diseases such as diabetes. A recent review of all validation studies found that medical data in the GPRD were generally of high quality (Herrett et al., 2010).

PHARMO RLS links the use of prescription drugs (pharmacy database, including dispensed drug, dispensing date, amount dispensed, and written dosage instructions) and diagnostic/therapeutic data from hospitals to the same patient. Currently, data are collected from a population of about three million residents in the Netherlands and are representative of the total Dutch population. A previous study of PHARMO RLS data has shown a high level of data validity with respect to the reporting of hip fractures (>89% of fractures were confirmed; Herings et al., 1996).

In Denmark, separate registers of computerized medical records on all contacts to hospitals and on the use of drugs can be linked for the entire population. The Ministry of the Interior keeps records of all inhabitants, including their migrations and dates of birth and death. Information on hospital admissions comes from the National Hospital Discharge Register (Andersen et al., 1999), which covers all inpatient contacts from 1977 onward and from 1995 also all outpatient visits to hospitals, outpatient clinics, and emergency rooms. The register has an almost 100% capture of contacts and the validity of registrations is high (Mosbech et al., 1995). The Danish Medicines Agency keeps a nationwide register of all prescription drugs sold at pharmacies throughout the country from 1996 onward, the National Pharmacological Database (www.dkma.dk). All prescriptions are registered with ATC code, dosage, and date.

### Study populations

In all three registries, the exposed cohort consisted of all patients (aged 18+) with at least one prescription of antidiabetic (AD) medication during valid data collection (GPRD: between 1987 and 2010, PHARMO: 1998 and 2008, Denmark: 1996 and 2007). The date of the first AD prescription within this period defined the index date. Patients with only a recorded prescription of insulin at their index date were excluded from the cohort. Therefore, all patients had a record of a non-insulin antidiabetic drug (NIAD) prescription on their index date. NIAD drug users were matched by year of birth and sex to control persons, who did not have an AD (NIAD or insulin) prescription any time during follow-up. In GPRD, one control was matched to each exposed patient, in PHARMO four controls and in Denmark three controls per patient. Controls were assigned the same index date as their matched NIAD drug user. All participants were followed from the index date to the end of data collection, the patient’s transfer out of the registry, emigration, or the patient’s death, whichever came first. The study populations from PHARMO and Denmark have been described before (Bazelier et al., 2012a,b).

### Exposure

The total period of follow-up for each patient was divided into periods of current exposure and past exposure, with patients moving between current and past use. In GPRD, each period of current exposure started with an AD prescription and ended 3 months later, or on the date that a new AD drug was prescribed within this period. In PHARMO and the Danish registries, each period of current exposure started with an AD prescription and ended 3 months after the expected duration of AD therapy, or on the date that a new AD drug was prescribed within this period. In PHARMO, the expected duration of NIAD therapy was based on the total quantity and the prescribed daily dose. In case of missing data, the median expected duration of treatment (based on data of other NIAD prescriptions) was used. For insulin treatment, the median time between two insulin injections (based on all insulin prescriptions) was taken as the expected duration of use. In Denmark, the expected duration of NIAD therapy was defined as the median time between two NIAD prescriptions, based on all NIAD prescriptions. For insulin treatment, the median time between two insulin injections (based on all insulin prescriptions) was taken as the expected duration of use.

At the start of each interval, each patient was classified as a current user of AD medication if they had an AD prescription on that start date or in the 3 months before. The current user status was determined for five different groups of AD medication: biguanides, sulfonylureas, TZDs, insulin, or other ADs (including Dipeptidyl Peptidase 4 inhibitors, glinides, Glucagon-Like Peptide-1 analogs, and alpha glucosidase inhibitors). For control persons, the total period of follow-up was divided into periods of 6 months.

### Study outcomes

Patients were followed up for the occurrence of a first fracture. The types of fracture were classified according to the International Classification of Diseases (ICD-10) categories. These included S02, S12, S22, S32, S42, S52, S62, S72, S82, S92, T02, T08, T10, and T12. An osteoporotic fracture was defined as a fracture of the radius/ulna, vertebrae, femur, hip, humerus, pelvis, or ribs.

### Statistical analysis

Incidence rates of fractures were estimated for diabetic patients and for controls. We computed person-years of follow-up by adding all person-time from the start date to either the date of first fracture or to the date of censoring, if no fracture had occurred. The incidence rate of fracture was defined as the number of fractures per 1,000 person-years. To study patient characteristics of TZD users, we randomly selected one TZD prescription for every patient who ever used a TZD during follow-up.

We compared risks of fracture in current users of TZDs with that in users of other AD medication, using a time-dependent Cox proportional hazards analysis. The analyses were adjusted for age and sex, and fracture risks were stratified by fracture type and sex. First, this was done for every registry separately. Then, we combined all records in an individual patient data meta-analysis and estimated fracture risks, thereby also adjusting for the origin of the data (i.e., GPRD, PHARMO, or Denmark). In a fully adjusted analysis we additionally adjusted for current use of other AD medication.

To investigate the risk of fracture in prolonged users of TZDs, we stratified fracture risks in current TZD users by number of TZD prescriptions ever before (using the multi-country dataset). Wald tests were used to examine if there were significant differences between differently exposed groups.

As a sensitivity analysis, we combined fracture risk estimates of the three separate studies in an inverse-variance fixed effect meta-analysis (Parmar et al., 1998). Heterogeneity statistics were derived. All data management and statistical analyses were conducted using SAS^®^ 9.1/9.2 and Review Manager^®^ 5.1 software.

## Results

Our study populations included 196,024 diabetic patients from the GPRD, 123,452 patients from PHARMO, and 180,049 patients from Denmark. Their mean age at index date (first NIAD prescription) varied between 63 and 65 years. PHARMO comprised slightly more female patients (53%) than the GPRD (45%) and the Danish registries (47%). The mean duration of follow-up after the index date was approximately 5 years for diabetic patients from all three registries. The GPRD was the only database in which information on smoking and body mass index was captured. Further baseline characteristics are shown in Table 1.

**Table 1:** **Baseline characteristics**.

	GPRD		PHARMO		Danish registries	
	Diabetic patients	Controls	Diabetic patients	Controls	Diabetic patients	Controls
	*n* = 196,024	*n* = 196,024	*n* = 123,452	*n* = 451,388	*n* = 180,049	*n* = 490,147
Mean duration of follow-up after index date, yrs (range)	4.7 (0.0–21.4)	4.6 (0.0–21.5)	4.5 (0.0–11.0)	4.0 (0.0–11.0)	5.3 (0.0–12.0)	6.2 (0.0–12.0)
Sex female	45.1%	45.1%	52.5%	52.9%	47.0%	47.4%
Mean age at index date	65.0	65.0	64.0	64.0	62.6	62.5
**BMI**						
<20	1.5%	4.5%				
20–25	14.7%	28.9%				
25–30	34.0%	32.3%				
>30	44.5%	15.9%				
Unknown	5.4%	18.4%				
**SMOKING**						
Never	41.1%	41.8%				
Current	17.8%	18.8%				
Ex	32.3%	22.1%				
Unknown	8.8%	17.4%				
**COMORBIDITY 1 YEAR BEFORE (a)**						
Fracture	1.2%	1.2%	0.5%	0.6%	2.4%	2.3%
Congestive heart failure	1.6%	0.7%	0.1%	0.1%	0.6%	0.1%
Rheumatoid arthritis	0.3%	0.3%	0.1%	0.1%	0.3%	0.2%
Cerebrovascular disease	2.2%	1.1%	0.9%	0.5%	2.6%	0.9%
Inflammatory bowel disease	0.2%	0.1%	0.2%	0.1%	0.4%	0.2%
Epilepsy	0.5%	0.5%	0.1%	0.1%	0.3%	0.2%
Diabetic retinopathy	2.8%	0.0%	0.0%	0.0%	0.9%	0.0%
**DRUG USE 1 YEAR BEFORE (a)**						
Statins	36.5%	12.4%	26.1%	13.9%	16.9%	6.3%
Antidepressants	15.7%	10.4%	10.0%	7.7%	12.2%	8.3%
Antipsychotics	2.9%	1.5%	3.1%	2.2%	5.7%	2.9%
Anxiolytics/hypnotics	10.4%	8.4%	23.6%	20.8%	21.1%	17.4%
Anticonvulsants	3.0%	2.2%	2.7%	2.3%	2.6%	1.9%
Opioids	4.9%	2.9%	7.6%	5.6%	13.5%	8.7%
Oral glucocorticoids	6.8%	4.4%	10.7%	6.7%	8.3%	5.0%
Bisphosphonates	2.0%	2.5%	3.0%	3.3%	1.0%	1.4%
Estrogen	1.2%	1.9%	2.5%	3.0%	4.5%	5.5%
Calcium	0.5%	0.5%	2.9%	3.3%	0.9%	0.9%
Vitamin D	2.7%	2.8%	0.8%	0.8%	0.1%	0.1%
Thiazide diuretics	20.9%	13.2%	8.4%	4.4%	19.3%	10.1%

*(a) Within people with more than 1 year of data collection*.

During the study period, 19.5% of the diabetic patients from the GPRD had been prescribed a TZD at least once (Table 2). For PHARMO, this was 12.2% and for the Danish registries 4.2%. Incidence rates of fracture were much higher in Denmark (27.4 fractures per 1,000 person-years for diabetic patients) than in the GPRD and PHARMO (with incidence rates of 10.3 and 7.9, respectively). The difference in incidence rates between diabetic patients and controls was most pronounced in Denmark as well.

**Table 2:** **Use of antidiabetic medication and incidence rate of fractures during follow-up**.

	GPRD		PHARMO		Danish registries	
	Diabetic patients	Controls	Diabetic patients	Controls	Diabetic patients	Controls
	*n* = 196,024	*n* = 196,024	*n* = 123,452	*n* = 451,388	*n* = 180,049	*n* = 490,147
**ANTIDIABETIC DRUG USE-ANY TIME DURING FOLLOW-UP**						
Biguanide	83.7%	–	80.8%	–	68.2%	–
Sulfonylureum	62.4%	–	70.1%	–	76.3%	–
Thiazolidinedione	19.5%	–	12.2%	–	4.2%	–
Insulin	13.1%	–	21.3%	–	23.6%	–
Other	8.9%	–	3.5%	–	9.9%	–
**INCIDENCE RATE OF FRACTURE (a)**						
Any fracture	10.33	9.94	7.92	7.42	27.36	23.90
Osteoporotic fracture	5.82	5.85	6.34	5.96	17.76	15.85
Hip	1.66	1.53	3.94	3.70	6.72	5.46
Vertebral	0.48	0.52	0.39	0.36	1.45	1.20
Radius/ulna	1.46	1.77	0.48	0.59	4.63	5.45
Femur	0.28	0.25	0.27	0.24	0.67	0.49
Pelvis	0.29	0.27	0.37	0.34	0.63	0.60
Humerus	1.19	1.06	0.56	0.47	3.48	2.56
Ribs	0.56	0.53	0.32	0.27	0.92	0.75
Patella	0.11	0.10	0.09	0.07	0.32	0.28
Tibia/fibula	0.47	0.36	0.32	0.28	2.71	2.00
Ankle	0.86	0.69	0.62	0.48	2.01	1.39
Foot	0.73	0.67	0.10	0.07	1.83	1.52

*(a) Number of fractures per 1,000 person-years*.

Table 3 shows that the GPRD comprised more prolonged users of TZDs (with a mean amount of 14 TZD prescriptions before) than the other two registries (6–8 TZD prescriptions before). In the GPRD, insulin was less often prescribed together with a TZD than in PHARMO and Denmark, but insulin use was overall lower in the GPRD (Table 2).

**Table 3:** **Characteristics of current TZD users, based on one random prescription per patient**.

	GPRD	PHARMO	Danish registries
	Random TZD prescriptions	Random TZD prescriptions	Random TZD prescriptions
	*n* = 38,438	*n* = 15,118	*n* = 7,603
Sex female	42.9%	52.1%	42.7%
Mean age at prescription date	64.3	63.2	61.6
Mean number of TZD prescriptions before	14.2	6.3	7.6
Mean duration of follow-up after index date, yrs	4.2	3.8	5.5
**CURRENT USE OF**			
Biguanide	77.5%	66.2%	85.2%
Sulfonylureum	53.7%	54.4%	47.2%
Insulin	5.2%	10.2%	9.4%
Other	4.7%	1.8%	6.4%

Table 4 shows that current TZD users had an increased risk of fracture compared with patients who were exposed to other AD drugs in GPRD, in PHARMO, but not in Denmark. In all three registries, the risk of fracture was however increased for women: hazard ratios (HR) 1.48 (1.37–1.60) in GPRD, HR 1.35 (1.15–1.58) in PHARMO, and HR 1.22 (1.03–1.44) in Denmark. TZD users from the GPRD had an increased risk for fractures of the radius/ulna, pelvis, humerus, tibia/fibula, and ankle, compared with other AD drug users. In PHARMO and Denmark, numbers of fractures in TZD users were generally too low to conclude anything on specific fracture types. One consistent finding was that no increased risk of hip fracture was apparent for TZD users in any of the registries.

**Table 4:** **Risk of fracture in current TZD users compared with other antidiabetic users, by type of fracture and sex**.

	GPRD				PHARMO				Danish registries			
	Fracture, *n* =	Age-sex adj HR	CI		Fracture, *n* =	Age-sex adj HR	CI		Fracture, *n* =	Age-sex adj HR	CI	
Control (other AD drug user)	7,245	1			3,797	1			21,202	1		
**CURRENT TZD USER**												
Any fracture	1,196	1.33	1.25	1.41	235	1.22	1.07	1.39	213	1.03	0.90	1.18
Male	401	1.10	0.99	1.22	63	0.98	0.76	1.26	74	0.79	0.63	1.00
Female	795	1.48	1.37	1.60	172	1.35	1.15	1.58	139	1.22	1.03	1.44
Osteoporotic fracture	592	1.27	1.16	1.38	176	1.23	1.06	1.44	116	0.98	0.81	1.18
Male	166	0.99	0.84	1.17	45	1.03	0.76	1.39	37	0.78	0.57	1.09
Female	426	1.42	1.28	1.57	131	1.32	1.11	1.59	79	1.11	0.88	1.38
Hip	109	0.95	0.78	1.16	86	1.08	0.87	1.35	19	0.53	0.34	0.84
Vertebral	41	0.97	0.70	1.35	17	1.80	1.08	3.00	11	1.00	0.55	1.81
Radius/ulna	189	1.59	1.35	1.86	21	1.49	0.94	2.35	43	1.23	0.91	1.67
Femur	20	1.09	0.68	1.74	11	1.87	1.00	3.52	2	0.49	0.12	1.99
Pelvis	31	1.47	1.00	2.16	8	1.06	0.52	2.17	3	0.82	0.26	2.55
Humerus	161	1.53	1.28	1.81	26	1.59	1.05	2.40	28	1.08	0.75	1.58
Ribs	54	1.07	0.80	1.43	7	0.76	0.35	1.64	11	1.38	0.76	2.52
Patella fracture	12	1.26	0.68	2.35	2	0.89	0.21	3.75	3	1.12	0.36	3.52
Tibia/fibula fracture	68	1.50	1.15	1.96	15	1.69	0.98	2.92	36	1.64	1.17	2.28
Ankle fracture	128	1.65	1.35	2.00	23	1.22	0.79	1.89	25	1.46	0.98	2.18
Foot fracture	91	1.18	0.94	1.48	4	1.46	0.51	4.18	27	1.52	1.03	2.23

Combining the data in an individual patient data meta-analysis resulted in a 1.3-fold increased risk of any fracture for current TZD users versus other AD drug users [fully adjusted (adj.) HR 1.27 (1.21–1.34), Table 5]. There was an increased risk for women [adj. HR 1.44 (1.35–1.53)], but not for men [adj. HR 1.05 (0.96–1.14)]. Risks were increased for fractures of the radius/ulna, humerus, tibia/fibula, ankle, and foot, but not for hip/femur or vertebral fractures.

**Table 5:** **Multi-country: risk of fracture in current TZD users compared with other antidiabetic users, by type of fracture and sex**.

	Multi-country						
	Fracture, *n*=	Age-sexadj HR	CI		Fully adj HR (a)	CI	
Control (other AD drug user)	32,244	1			1		
**CURRENT TZD USER**							
Any fracture	1,644	1.25	1.18	1.31	1.27	1.21	1.34
Male	538	1.01	0.92	1.10	1.05	0.96	1.14
Female	1,106	1.42	1.33	1.51	1.44	1.35	1.53
Osteoporotic fracture	884	1.20	1.12	1.29	1.23	1.14	1.32
Male	248	0.95	0.83	1.08	0.99	0.87	1.13
Female	636	1.35	1.25	1.47	1.37	1.26	1.49
Hip	214	0.91	0.79	1.05	0.93	0.81	1.06
Vertebral	69	1.08	0.84	1.39	1.12	0.87	1.44
Radius/ulna	253	1.47	1.29	1.68	1.50	1.31	1.71
Femur	33	1.11	0.78	1.59	1.15	0.80	1.65
Pelvis	42	1.30	0.94	1.79	1.32	0.96	1.82
Humerus	215	1.48	1.28	1.71	1.53	1.32	1.77
Ribs	72	1.06	0.83	1.35	1.11	0.87	1.43
Patella fracture	17	1.09	0.65	1.80	1.13	0.68	1.87
Tibia/fibula fracture	119	1.56	1.28	1.89	1.60	1.32	1.95
Ankle fracture	176	1.53	1.30	1.80	1.57	1.34	1.85
Foot fracture	122	1.24	1.02	1.51	1.32	1.08	1.60

*(a) Adjusted for age, sex, current use of biguanides, sulfonylureas, insulin, or other antidiabetics (including DPP-4 inhibitors, glinides, GLP-1 analogs, and alpha glucosidase inhibitors)*.

Table 6 presents the stratification of current TZD users by the number of TZD prescriptions they had before. Current TZD users with more than 25 TZD prescriptions ever before had a 1.6-fold increased risk of fracture compared with other AD drug users [HR 1.59 (1.46–1.74)]. When we stratified the overall fracture risk to risk of fractures of the extremities for women who had been prescribed a TZD more than 25 times ever before, we found a doubled increased fracture risk. For men, the risk of fractures of the extremities increased from HR 1.15 (0.95–1.38) in the lowest exposure group (1–10 prescriptions) to HR 1.31 (1.05–1.64) in the group with largest exposure (>25 prescriptions), but differences between these groups were not statistically significant.

**Table 6:** **Multi-country: risk of fracture in current TZD users compared with other antidiabetic users, by number of TZD prescriptions**.

	Multi-country								
	Fracture, *n*=	Age-sex adj HR	CI			Fully adj HR (a)	CI		
No TZD	32,244	1				1			
**CURRENT TZD**									
Any fracture	1,644	1.25	1.18	1.31		1.27	1.21	1.34	
Number of TZD prescriptions ever before
1–10	590	1.09	1.00	1.18		1.11	1.02	1.21	
11–25	516	1.22	1.11	1.33	(c)	1.24	1.13	1.35	(c)
>25	538	1.56	1.43	1.70	(d)	1.59	1.46	1.74	(d)
Fracture of extremities (b)	981	1.45	1.35	1.55		1.49	1.39	1.59	
Male	312	1.20	1.07	1.36		1.25	1.10	1.41	
Female	669	1.60	1.47	1.73		1.64	1.51	1.78	
Number of TZD prescriptions ever before
1–10	227	1.35	1.18	1.55		1.38	1.21	1.58	
11–25	214	1.59	1.39	1.83	(c)	1.63	1.42	1.87	(c)
>25	228	1.99	1.74	2.29	(d)	2.06	1.79	2.36	(d)

*(a) Adjusted for age, sex, current use of biguanides, sulfonylureas, insulin, or other antidiabetics (including DPP-4 inhibitors, glinides, GLP-1 analogs, and alpha glucosidase inhibitors)*.

*(b) Fracture of the tibia/fibula, ankle, foot, radius/ulna, humerus, wrist*.

*(c) Statistically significant difference between 11–25 and >25 prescriptions, based on Wald test*.

*(d) Statistically significant difference between 1–10 and >25 prescriptions, based on Wald test*.

**Figure 1:**
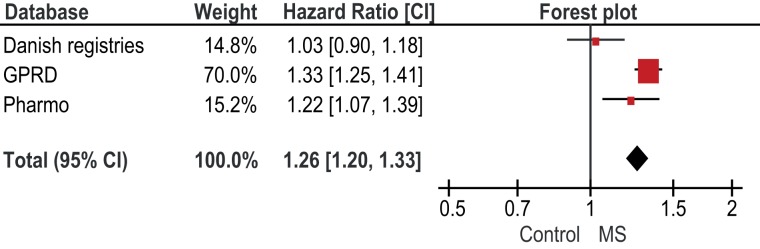
**Forest plot of age-sex adjusted Hazard Ratios for any fracture**.

A sensitivity analysis showed that a traditional meta-analysis (presented in Figure 1) provided similar results as the results from Table 5. For any fracture, the pooled age-sex adjusted estimate of the individual studies was HR 1.26 (1.20–1.33). Overall, there was significant heterogeneity between the studies (*p* = 0.003). However, for women only the heterogeneity statistic was not significant (*p* = 0.08). For men only, the heterogeneity statistic was significant (*p* = 0.04).

## Discussion

In this study, we consistently found a 1.2- to 1.5-fold increased risk of fractures for women using TZDs, but not for men, across three different healthcare registries.

Our findings are comparable to clinical trial results. A meta-analysis capturing 10 randomized controlled trials reported that patients who were using TZDs experienced a 1.5-fold increased risk of fracture compared with patients using other AD drugs [OR 1.45 (1.18–1.79)]. Another recent trial that was not incorporated in this meta-analysis showed a similar result [RR 1.57 (1.26–1.97); Home et al., 2009]. Both the meta-analysis and the more recent trial found a significantly increased risk of fractures among women but not among men, which is in line with our findings. Another similarity is the finding of an excess risk of distal fracture events.

Observational studies have also evaluated the association between TZD use and fracture risk, providing some contrasting results (Betteridge, 2011). Meier et al. (2008) performed a nested case-control study and found that the receipt of more than eight TZD prescriptions was associated with an OR for fracture of 2.43 (1.49–3.95) versus no use of TZDs. The increased risk was found for hip/femur, humerus, and wrist/forearm fractures and there was a similar increase in women and men. From their prospective cohort study, Dormuth et al. (2009) concluded that treatment with a TZD was associated with a 1.3-fold increased risk of peripheral fractures compared with treatment with a sulfonylureum [HR 1.28 (1.10–1.48)], regardless of sex. Habib et al. (2010) reported in their cohort study that TZD users had a 1.4-fold increased risk of fracture [HR 1.35 (1.05–1.71)]. Risks were increased for women, but not for men, and for extremity fractures, but not for fractures of the femur or vertebrae. Using a self-controlled case series design, Douglas et al. (2009) found a within-person rate ratio of 1.43 (1.25–1.62) for fracture comparing exposed with unexposed periods among patients prescribed any TZD. Findings were similar in men and women and the increased risk was evident at a range of fracture sites, including hip, spine, arm, foot, wrist, or hand. However, a recent study that evaluated the performance of different study designs found that within-person study designs had lower precision and greater susceptibility to bias than cohort studies (Nicholas et al., 2012). Aubert et al. (2010) showed in their cohort that TZD treatment was associated with an increased risk of fracture in both men and women. The estimated risk of fracture was nevertheless higher in women than in men.

The different conclusions that are reached by observational studies may thus be explained by the choice of different study designs, but also by features of the registries that are used, differences in prescribing habits between countries, and other differences in patient characteristics or medication use. In our study, we therefore explored heterogeneity between the three registries. Several differences were observed. Fracture incidences were much higher in Denmark than in the other two registries. This may be due to higher incidence rates for fracture in Denmark (Kanis et al., 2002), but also to more complete recording of all different fracture types. From 1995 onward, inpatient and outpatient diagnoses are captured in the Danish registries, while PHARMO only comprises information on inpatient diagnoses. This probably explains the low incidence rates for fractures of the extremities in PHARMO. The proportion of patients who were prescribed a TZD was lower in Denmark (4.2%) than in the GPRD (19.5%) and PHARMO (12.2%). This may be caused by different marketing strategies for TZDs in these countries, or by prescribing preferences of the physicians. The use of AD co-medication also differed for TZD users from the separate registries. For example, the use of insulin was less common in users from the GPRD than from the other two registries.

Despite all these considerations and differences between the three countries, we actually found a fair amount of similarities in outcomes when using the same study design. Firstly, fracture risks for TZD users were only increased in women, not in men. Secondly, there was a tendency for an increased risk of distal fractures, although the number of fractures in PHARMO and Denmark was much lower than in GPRD (because the GPRD captured more TZD users). Combining these three datasets in an individual patient data meta-analysis increased the total number of fractures and therefore enabled us to stratify risks to various relevant subgroups. These included different fracture types, sex, and duration of use. It was even possible to stratify the risk of fracture in such a way that all risk factors were combined: we estimated the risk of fractures of the extremities in women, who were prolonged users of TZDs. The estimate was significantly different from other exposed groups.

The increased risk of fracture that we found may be caused by different mechanisms. TZDs have been shown to increase the expression of adipogenesis at the expense of osteoblastogenesis through activation of PPARγ (Benvenuti et al., 2007; Grey, 2008). However, this does not explain why fracture risks would only be increased in women and not in men. A possible explanation involves the finding that PPARγ ligands can stimulate the differentiation of a number of cell types and not only those of mesenchymal origin: Rubin et al. (2000) have demonstrated that PPARγ ligands inhibit the expression of aromatase and hence estrogen biosynthesis in adipose tissue. This may increase estrogen deficiencies in menopausal women and thereby lead to increased bone resorption and loss of trabeculae. Another recent study found that TZD exposure *in vitro* may stimulate adipogenesis but does not directly alter osteoblast differentiation, mineralization, or lineage commitment from human bone marrow stromal cells (Beck et al., 2012). Furthermore, TZDs may activate PPARγ in tissues other than bone, such as the hypothalamus-pituitary-gonad axis to indirectly regulate bone mass (Wei and Wan, 2011).

The specific risk of fractures of the extremities with the use of TZDs is not fully understood. It has been demonstrated that rosiglitazone administration in mice causes a loss of bone mass in cortical bone, possibly through a decrease in bone formation expressed by decreased bone alkaline phosphatase (Broulík et al., 2011). Another explanation may be a decrease in blood flow to the extremities that causes an increase in the rate of bone loss (Vogt et al., 1997).

Besides the use of TZDs, T2DM has also been associated with adverse effects on the skeleton (Janghorbani et al., 2007; Vestergaard, 2007). Possible mechanisms include direct effects of the high glucose levels on bone turnover (Williams et al., 1997), increased urine calcium loss (McNair et al., 1979), and changes in vitamin D metabolism (Bouillon, 1991). Complications of diabetes, such as neuropathy and angiopathy, may also contribute to an increased risk of fracture.

There were several advantages to this multi-country study. A major advantage of our study was the availability of individual patient data from three different healthcare registries. In total, we had information of more than 60,000 patients who had used a TZD. There was detailed longitudinal information on drug prescribing. But there are important limitations. HRs were adjusted for age, sex, and the use of AD medication other than TZDs, but not for other potential risk factors. In our previous studies from PHARMO and the Danish registries, adjustments for other potential confounders did, however, not substantially change the findings (Bazelier et al., 2012a,b). The increased fracture risk that we found for prolonged users of TZDs may be caused by the drug but also by the underlying disease. By adjusting our analyses for the use of AD medication other than TZDs, we indirectly adjusted for the severity of the underlying disease. Issues with latency of osteoporosis may have led to protopathic bias in our studies; although not registered in the databases, osteoporosis may already have been noticed by a physician, which may have stopped him from prescribing a TZD. The true associations between TZD use and the risk of fracture may therefore be greater than reported in our studies.

In conclusion, we found a 1.3-fold increased risk of fracture for current TZD users versus users of other AD drugs, using an individual patient data meta-analysis. The risk of fracture with current TZD use was increased in women, but not in men. TZD users had an increased risk for fractures of the extremities, and risks further increased for prolonged users of TZDs.

## Conflict of Interest Statement

The Department of Pharmacoepidemiology and Clinical Pharmacology, Utrecht Institute for Pharmaceutical Sciences, has received unrestricted research funding from the Netherlands Organisation for Health Research and Development (ZonMW), the Dutch Health Insurance Board (CVZ), the Royal Dutch Association for the Advancement of Pharmacy (KNMP), the private-public funded Top Institute Pharma (www.tipharma.nl), includes co-funding from universities, government, and industry), the EU Innovative Medicines Initiative (IMI), EU seventh Framework Program (FP7), the Dutch Medicines Evaluation Board, the Dutch Ministry of Health and industry (including GlaxoSmithKline, Pfizer, and others). Tjeerd van Staa and Arlene Gallagher also work for the Clinical Practice Research Datalink (CPRD), UK, which operates within the Medicines and Healthcare products Regulatory Agency (MHRA). CPRD has received funding from the MHRA, Department of Health, Wellcome Trust, Medical Research Council, NIHR Health Technology Assessment programme, Innovative Medicine Initiative, UK Department of Health, Technology Strategy Board, Seventh Framework Programme EU, various universities, contract research organisations, and pharmaceutical companies.
